# Active Purchasing: Empirical Insights From the Dutch Healthcare System and Lessons for Low- and Middle-Income Countries: Comment on "Measuring Active Purchasing in Healthcare: Analysing Reallocations of Funds Between Providers to Evaluate Purchasing Systems Performance in the Netherlands"

**DOI:** 10.34172/ijhpm.9169

**Published:** 2025-08-04

**Authors:** Xiaohui Hou, Yanfang Su

**Affiliations:** ^1^Health, Nutrition, and Population Global Practice, World Bank Group, Washington, DC, USA.; ^2^Department of Global Health, School of Public Health, University of Washington, Seattle, WA, USA.; ^3^Evans School of Public Policy and Governance, University of Washington, Seattle, WA, USA.

**Keywords:** Active Purchasing, Strategic Purchasing, Managed Competition, Resource Allocation

## Abstract

Stadhouders et al critically examines the assumptions behind managed competition, revealing that competitive systems alone may not drive efficiency gains through fund reallocation. Their findings from the Dutch hospital sector suggest limited or low reallocation of funds between providers and highlight the need for monitoring resource allocation progress, understanding barriers and adjusting incentives for better functioning healthcare markets. For low- and middle-income countries (LMICs) undergoing health reforms, the Dutch experience underscores the importance of tailoring purchasing models to local contexts. LMICs should enhance data use for more strategic decision-making as well as building regulatory framework and institutional capacity for stronger implementation. Future research should explore how purchasing models interact with diverse health system characteristics to inform system-specific reforms.

## Background

 Stadhouders et al^1^ provide an important empirical analysis of the effectiveness of managed competition in the Dutch healthcare system. Their use of the Market Activity Index as a measure of active purchasing offers a novel approach to evaluating how funds are reallocated between providers. The study’s key strength lies in its cross-sectoral comparison of purchasing systems, allowing for insights into the effectiveness of managed competition relative to other models, such as a single-payer system for long-term care or a single-payer system for social care services. The study’s central finding that competitive managed care reforms did not result in higher fund reallocations compared to non-competitive systems raises critical questions about the effectiveness of active purchasing, or managed competition, in achieving intended allocative efficiency.

 These findings align with previous research suggesting that managed competition does not always lead to substantial efficiency gains.^2^ While managed competition theoretically encourages purchasers to actively purchase care from well-performing providers, empirical evidence remains mixed regarding its effectiveness in driving meaningful improvements in healthcare resource allocation. A narrative review with 42 studies suggested that in addition to the design and implementation of four core functions of purchasing (benefits specification, contracting arrangements, provider payment, and performance monitoring), the enabling environment (both economic and political), and the level of development of the country’s health system are also relevant. Stadhouders et al focuses on a high-income country (HIC)with a well-developed insurance-based system. However, their findings carry implications for low- and middle-income countries (LMICs), where healthcare purchasing mechanisms are often still developing. Many LMICs are shifting from passive to strategic purchasing to improve efficiency and equity.^3^ However, as Stadhouders et al demonstrate, even in a highly regulated and structured healthcare system like that of the Netherlands, active purchasing does not necessarily result in increased allocative efficiency. This raises important considerations for LMICs seeking to strengthen their healthcare through purchasing mechanisms.

 First, some HICs have insurance-based systems, but others operate national health systems supported by general taxation. For example, countries like Germany and Japan rely on mandatory social health insurance with multiple insurers and structured risk pooling, while systems in the UK are largely tax-funded, with health services primarily delivered by public providers. Therefore, it is important to be reminded that managed competition is not the only way for better or more efficient resource allocation.

 Second, although HICs often benefit from institutionalized purchasing and relatively well-developed financial management systems in both the public and private sectors, LMICs frequently face challenges such as underfunded public health systems, weak purchasing arrangements, and fragile public financial management. In addition, many LMICs rely heavily on out-of-pocket payments and fragmented financing mechanisms, including donor funding and limited public budgets.

 The question is how LMICs can use active or strategic purchasing to improve health resource allocation in their own country context.

## Active Purchasing and Strategic Purchasing

 As this commentary is to explore the implications of these empirical findings from Stadhouders et al for LMICs, it will be useful to review the concepts of active purchasing and strategic purchasing. The latter term is often used in LMIC contexts, while active purchasing is used in the context of Dutch and American healthcare system, particularly in the discussions on managed competition and market force. Statehooders et al define “active purchasing (c.q. strategic purchasing, contracting, commissioning, and procurement) as interventions by third-party payers to improve market outcomes.” While active purchasing can encompass tools such as contracting and commissioning, it is in principle fully aligned with the concept of strategic purchasing, as defined by Figueras and colleagues.^4^ The authors characterize strategic purchasing as a process that “aims to increase health systems’ performance through effective allocation of financial resources to providers.” This involves three sets of explicit decisions: “(*i*) which interventions should be purchased in response to population needs and wishes, taking into account national health priorities and evidence on cost-effectiveness; (*ii*) how [interventions] should be purchased, including contractual mechanisms and payment systems; and (*iii*) from whom [to purchase], in light of relative levels of quality and efficiency of providers.”^4^

 While the terms active purchasing and strategic purchasing are often used interchangeably, they carry slightly different emphases depending on the contexts—especially when comparing market-driven models (like managed competition) with governance-driven models (like those led by ministries or public purchasers).

 Strategic purchasing is a broad concept that refers to how health funds are allocated to providers in a way that aligns with health system goals—such as efficiency, equity, and quality. It emphasizes the functions of purchasing as described above: deciding what services to buy, from whom, and how to pay them. Strategic purchasing is not inherently market-driven—it can be implemented by public agencies or insurance funds, and it often involves strong governance, regulation, and alignment with national health priorities.

 Active purchasing, especially in the context of managed competition, leans more towards a market-oriented approach. The assumption is that competition among purchasers and providers will drive efficiency and responsiveness. In this model, purchasing is “active” in the sense that insurers or purchasers selectively contract providers, negotiate prices, and manage utilization.

## Strengthening Purchasing in Low- and Middle-Income Countries

 Transitioning from passive to strategic purchasing is a key challenge for many LMICs. Many countries still rely on financial flows that are determined by historical budgets rather than provider performance. Stadhouders et al suggest that merely introducing managed competition may not automatically improve efficiency. Instead, LMICs must focus on strengthening strategic purchasing mechanisms, which include performance-based financing that rewards efficiency. Empirical studies from LMICs, including studies of Ghana’s National Health Insurance Scheme and Thailand’s Universal Coverage Scheme, demonstrate that moving towards strategic purchasing can enhance provider accountability and cost efficiency.^5,6^ However, implementing these mechanisms requires stronger governance structures to prevent corruption and inefficiencies.

 The structure of the healthcare market also plays a crucial role in determining the success of active purchasing. A major question raised by Stadhouders et al is whether the Dutch hospital market is truly competitive, given the high level of provider concentration and consolidation. This issue is even more pronounced in LMICs, where healthcare markets are often fragmented in urban areas but monopolistic in rural regions. To improve efficiency through strategic purchasing, LMICs could benefit from diversifying their pool of healthcare providers across sectors. Relying solely on public facilities may limit access, especially in underserved areas. Engaging private providers such as hospitals, clinics, and diagnostic centers, can help fill critical gaps and improve sector competition. In addition to the potential efficiency gains, fostering more diversified pool of providers and improving the public–private interface could also enhance equity and access.

 Purchasers should play a more proactive role in influencing providers, rather than relying solely on market competition. By strategically selecting and contracting health service providers based on criteria such as capacity and geographical distribution, purchasers can ensure more equitable and efficient service delivery. Additionally, they can actively drive quality improvement through mechanisms like standardized treatment guidelines and robust monitoring systems. Incorporating financial incentives tied to quality enhancement can further encourage providers to prioritize better patient outcomes and adherence to best practices.

 Measuring performance in healthcare systems requires a broader approach than simply tracking efficiency through fund reallocations. While Stadhouders et al use the Market Activity Index to assess how funds shift between providers, LMICs should consider additional indicators that provide a more comprehensive picture of performance. In addition to quality, equity and financial risk protection indicators should be incorporated to ensure that purchasing reforms do not disproportionately benefit wealthier populations while leaving vulnerable groups behind. For instance, Kenya’s health-purchasing reforms have improved access, quality of care, and financial risk protection to some extent, though Kenya’s purchasing function needs further strengthening in many areas.^7^

## Challenges and Options in LMIC Contexts

 Strategic purchasing is shaped by a country’s financial and administrative capacity. Countries with greater fiscal space and stronger institutions are typically better equipped to design and implement effective purchasing arrangements. The literature highlights that resource availability influences not only which services are purchased, but also from whom they are purchased and under what contractual and regulatory conditions.^8^ Various approaches to health revenue mobilization and pooling—whether through general taxation, contributory insurance, or hybrid models—significantly influence the scope for strategic purchasing. This commentary argues that LMICs should aim to diversify their mix of healthcare providers to expand access and improve responsiveness. However, it is important to recognize that active purchasing alone does not automatically lead to more efficient or equitable resource allocation. To achieve these goals, complementary mechanisms are essential—such as innovative provider payment methods, robust performance monitoring, and strong regulatory oversight.

 Many LMICs face fundamental barriers to effective implementing strategic purchasing: weak information systems and regulatory frameworks are two salient examples. The findings from Stadhouders et al suggest that, even with the Netherlands’ strong institutions, selective contracting did not lead to significant reallocations. This raises concerns for LMICs, where information asymmetry is often a larger problem due to the lack of comprehensive data on provider quality and performance. Investing in robust health information systems is critical to enable data-driven purchasing decisions, as demonstrated by Rwanda’s performance-based financing model, which has linked payment incentives to improved health outcomes.^9^ Regulatory oversight also needs to be strengthened to prevent corruption and ensure that purchasing incentives align with quality and efficiency improvement goals. In addition, greater transparency in provider performance metrics is necessary to allow payers to make more informed decisions about which providers to contract with and reward for accessibility, efficiency and quality.

 In addition to these major challenges, limited technical capacity in areas such as health service costing, provider contracting, financial reporting and auditing, and broader accountability systems can significantly constrain the ability of a country to design and manage purchasing arrangements. Addressing these capacity gaps is essential to ensure that strategic purchasing can be implemented in a way that delivers value for money and advances health system goals.

## Conclusions

 The study by Stadhouders et al provides valuable empirical tests of the assumptions underlying managed competition. The findings suggest that competitive payer systems alone may not be sufficient to boost efficiency through fund reallocations. Given the evidence that the Dutch hospital sector may not be as competitive as assumed, policy-makers should consider additional tools to enhance active purchasing. The study raises important empirical and policy questions about the role of market structure in shaping purchasing behaviors. This has implications for many LMICs which are in the process of health reforms.

 The Dutch experience with managed competition highlights the need for LMICs to tailor their purchasing reforms to local contexts. Many LMICs have unique healthcare system structures, financing mechanisms, and levels of private-sector involvement that make direct replication of models from HICs impractical. As they consider leveraging market power through managed competition, LMICs should focus on building stronger strategic purchasing institutions that integrate performance-based incentives. While fostering competition where feasible, governments must also regulate providers to prevent inefficiencies and ensure cost-effectiveness. Enhancing data-driven decision-making through improved health information systems is critical to aligning purchasing decisions with quality improvements. Equity considerations should also be at the forefront of reform efforts to ensure that purchasing mechanisms do not exacerbate existing disparities in access to care. By taking these steps, LMICs can develop purchasing systems that not only improve allocative efficiency but also enhance the overall effectiveness and equity of their healthcare systems. In order to better inform policy decisions, future research should focus on how different purchasing models interact with health-system characteristics in LMICs.

## Ethical issues

 Not applicable.

## Conflicts of interest

 Authors declare that they have no conflicts of interest.
